# Supplementary material for: IGF1R Is a Potential New Therapeutic Target for HGNET-BCOR Brain Tumor Patients
Source: Int J Mol Sci. 2019 Jun 21;20(12):3027. doi: 10.3390/ijms20123027 (PMC6627083; doi:10.3390/ijms20123027)
Supplement: Supplementary file 1 [file ijms-20-03027-s001.pdf]

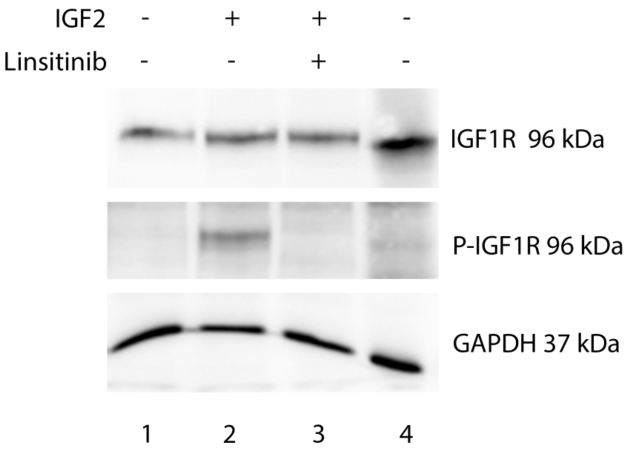

Figure S1. Lanes 1-3. Linsitinib inhibits the phosphorylation of IGF1R in PhKh1 cells. PhKh1 cells were stimulated after starvation with IGF2 in the presence or absence of linsitinib. The protein expression of IGF1R and its phosphorylated form (P-IGF1R) was analyzed by western blot with specific antibodies. Lane 4. IGF1R and P-IGF1R expression in the tumor material of P1. GAPDH was used as loading control.

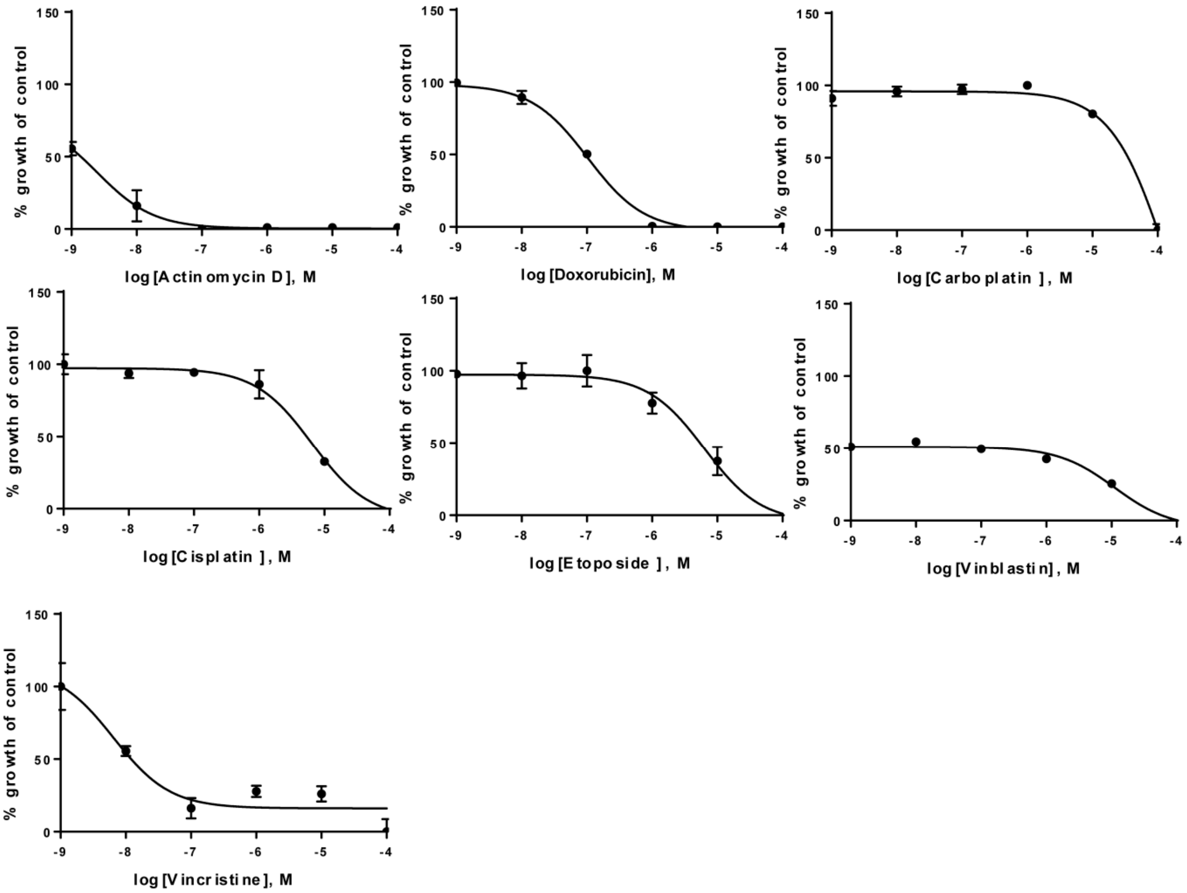

Figure S2. PhKh1 cells were treated with the indicated inhibitors from 1 nM to 100 μM. The logarithm of the molarity (M) is displayed on the X-axis. The percent of viable cells compared to the control treated with vehicle alone is shown on the Y-axis. The data were fitted to a sigmoidal dose-response curve using GraphPad software. A representative experiment of three independent experiments is shown.
